# A Rare Complication of HVAD Outflow Thrombosis and the Importance of HVAD Waveform Analysis

**DOI:** 10.1155/2019/6905397

**Published:** 2019-10-13

**Authors:** John Ning, Nunzio Gaglianello

**Affiliations:** Medical College of Wisconsin, 8701 West Watertown Plank Road Milwaukee, WI 53226, USA

## Abstract

We present a case of a 64-year-old female who was supported with an HVAD as bridge-to-transplant (BTT) who presented with a gastrointestinal (GI) bleeding and underwent esophagogastroduodenoscopy (EGD) and colonoscopy. Her waveforms changed abruptly following the procedure, and she decompensated. With various imaging modalities and hemodynamic monitoring, we felt that she had thrombus in her outflow graft, which improved following systemic heparinization. She was listed for cardiac transplantation and remained hospitalized. At the time of surgery, her outflow graft was noted to be compressed externally and pathology was consistent with platelet-fibrin thrombus deposition.

## 1. Introduction

In this case report, we describe a rare complication of outflow graft compression as well as our approach to analyzing HVAD waveform and use of imaging modalities that led to our differential diagnosis and management.

## 2. Case Presentation

64-year-old African American female with nonischemic cardiomyopathy implanted with an HVAD as a bridge to transplant, listed for cardiac transplantation as UNOS status 1B, had been doing well until the time of her hospital presentation. She was admitted to the hospital with concern for GI bleeding. Her hemoglobin was 7.9 g/dL, down from her baseline of 10.4 g/dL. Her lactate dehydrogenase (LDH) and haptoglobin were stable at presentation at 219 U/L and 64 mg/dL, respectively. The patient underwent evaluation for GI bleeding with an EGD and colonoscopy with no initial source found, so a capsule endoscopy was placed. On capsule endoscopy, the patient was found to have a jejunal bleeding. Following this, a push enteroscopy was performed with argon plasma coagulation and clipping of the arteriovenous malformation. After being transferred to the floor in a stable condition, the patient became acutely hypoxic and started having low flow alarms on her HVAD device and her waveform became less pulsatile. Her heart rate was in 120 s, mean arterial pressures were in 50 s, and she was transferred to a cardiovascular ICU for emergent intubation. She was started on a heparin drip as well as on vasopressors and inotropes for hemodynamic instability. After the transfer to the cardiovascular ICU, a bedside swan was obtained demonstrating the following hemodynamics: right atrium (RA) 13 mmHg, right ventricle 52/13 mmHg, pulmonary artery 53/28/40 mmHg, pulmonary capillary wedge pressure (PCWP) 28 mmHg, and cardiac output/cardiac index 3.0/1.6 L/min. Repeat lactate dehydrogenase and haptoglobin were 165 U/L and 48 mg/dL respectively.

## 3. Materials and Methods

**Figure 1:**
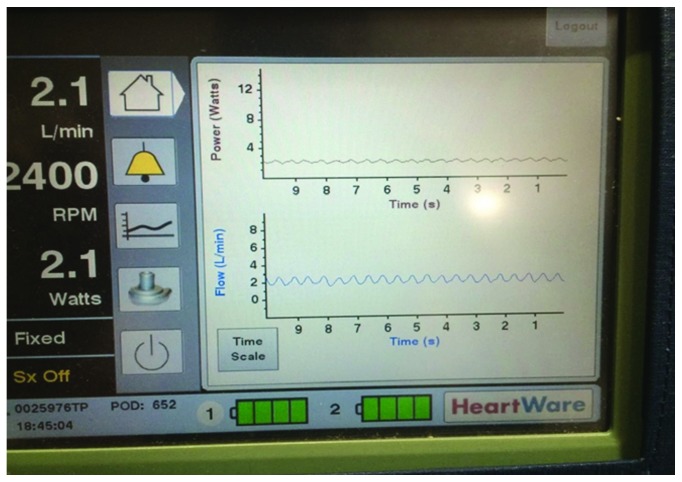
Demonstration of a low flow-low pulsatility waveform with no increase in HVAD power consumption.

**Figure 2:**
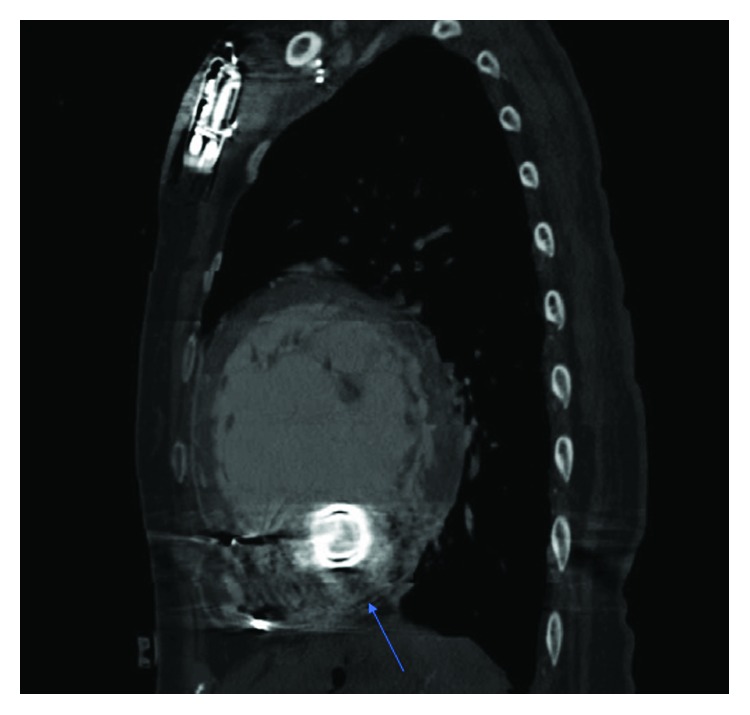
Gated CT scan of the chest with IV contrast in the sagittal plane demonstrating compression/thrombosis of the HVAD outflow graft (arrow).

The following are the pre-GI procedure HVAD parameters: flow 4.0 L/min, speed 2460 rpm, 2.8 watts, MAP 68 mmHg, and normal waveform pulsatility. The following are the post-GI procedure HVAD parameters: flow 2.1 L/min, speed 2400 rpm, and 2.1 watts (Figure 1). The transthoracic echocardiogram (TTE) showed a severely enlarged left ventricle with severe systolic dysfunction and severe global hypokinesis with an ejection fraction (EF) of 10-20% and moderate right ventricular systolic dysfunction. No inflow cannula thrombus was noted. Inflow cannula velocities were normal. Outflow cannula velocities were low at <1 m/s. After the patient was stabilized, a gated computed tomography (CT) of the chest demonstrated an interval development of nearly occlusive thrombus involving the proximal half of the outflow graft with opacification of the distal half (Figure 2).

## 4. Results

**Figure 3:**
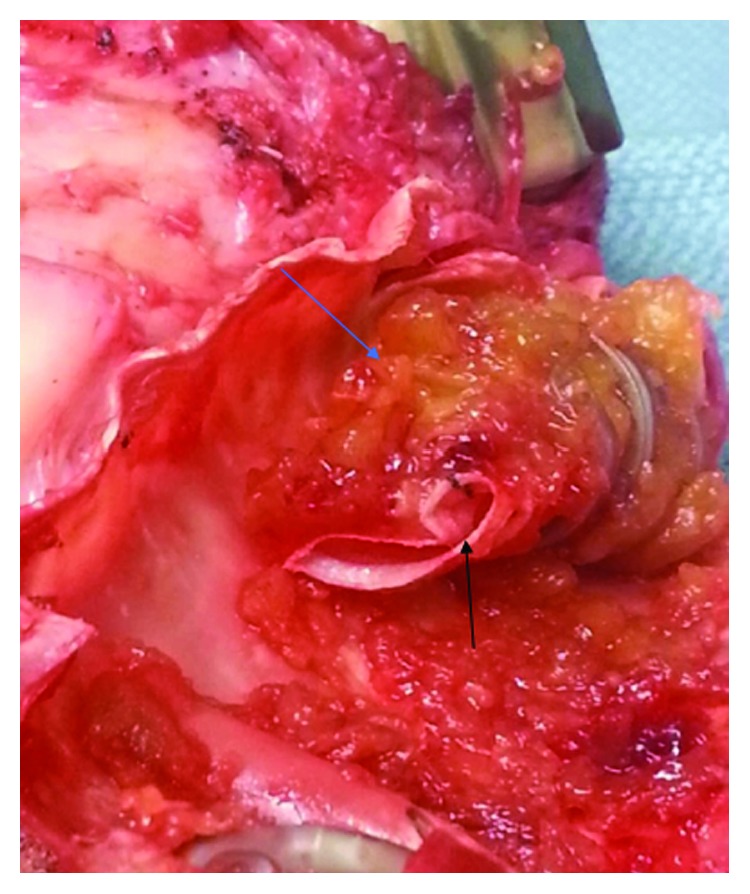
Analysis of the HVAD outflow graft at the time of cardiac transplantation demonstrating platelet-fibrin rich collection (blue arrow) causing external compression of the outflow graft (black arrow).

**Figure 4:**
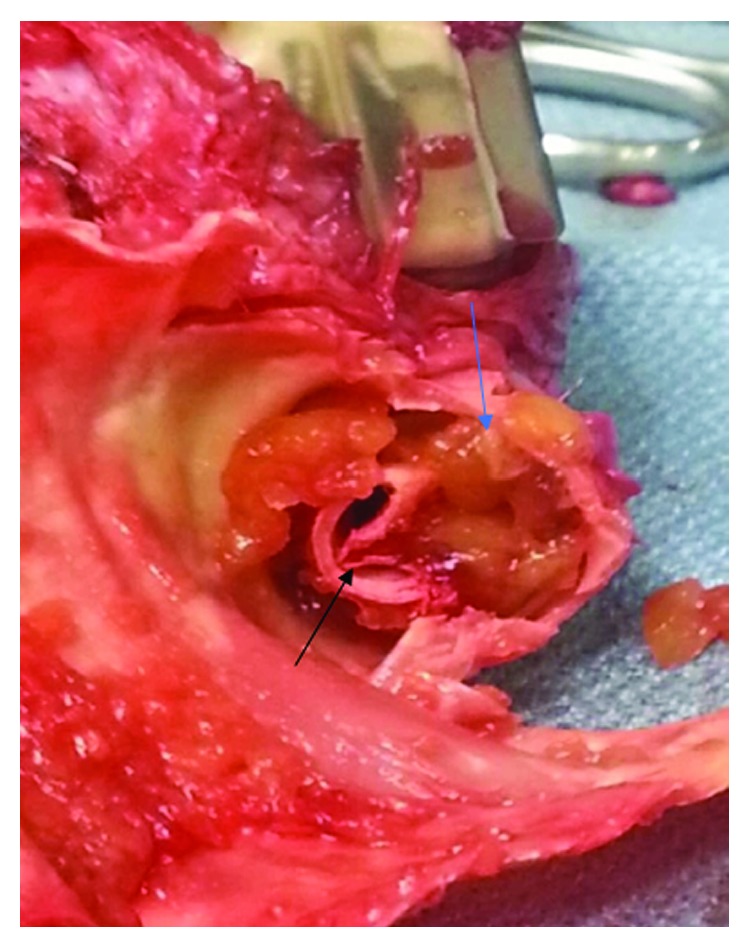
Different view of the HVAD outflow graft (black arrow) at the time of cardiac transplantation demonstrating external compression of the outflow graft by platelet-fibrin rich thrombus (blue arrow).

The patient's HVAD waveform improved with systemic heparinization. She was diuresed, extubated, and weaned off vasopressors and inotropes and was listed as UNOS status 1A and was successfully transplanted. At the time of transplantation and HVAD explantation, the outflow graft material was noted to be platelet-fibrin thrombus (Figures 3 and 4).

## 5. Discussion

Left ventricular assist devices (LVADs) serve as a bridge to transplant (BTT) and offer patients with advanced heart failure better survival and functional status and quality of life as they await heart transplantation [1]. Recently, the ENDURACE trial suggested that a small, intrapericardial, centrifugal flow LVAD was noninferior to axial flow LVAD in terms of survival free from disabling stroke or device removal for malfunction or failure [2].

A known serious complication of LVADs is the formation of thrombus within the pump impeller. Pump thrombus requiring exchange occurred at a rate of 0.04 events per patient year (EPPY) with a total suspected thrombus rate of 0.08 EPPY in patients implanted with an HVAD from the HeartWare BTT and CAP (Continued Access Protocol) trials [3]. This case is unique in that the thrombus formation was an external compression of fibrin-rich material that caused compression of the outflow graft. A recent study found that PTFE graft covering of the LVAD outflow graft can lead to graft occlusion and should be reconsidered a potentially harmful modification [4]. Our patient's LVAD was wrapped in polytetrafluoroethylene (PTFE) which does not breathe or leak. This leads to confinement of the outflow graft and potential external compression of the outflow graft limiting blood flow.

Interpreting the HVAD waveform and understanding changes in flows and pulsatility can help the clinician create a differential diagnosis. In this case, an abrupt change in pulsatility and flow led our group to suspect an inflow thrombus or outflow cannula thrombus/obstruction [5]. Other considerations in the setting of low flow-low pulsatility waveforms include right ventricular failure, cardiac tamponade, ventricular fibrillation, and rapid ventricular tachycardia. During the evaluation, an echocardiogram was obtained which did not demonstrate an inflow cannula thrombus or a pericardial effusion. The patient's hemodynamics from right heart catheterization were not consistent with right ventricle failure given that the RA pressure was only 13 mmHg as well as maintain a preserved PCWP : RA pressure ratio of 2 : 1 along with TTE findings of unchanged moderate RV systolic dysfunction.

Ultimately, a gated CT scan demonstrated compression of the outflow graft with narrowing of the outflow graft lumen. In the most common form of pump thrombosis, a clot forms on the pump rotor. This increased resistance manifests as an increase in pump power and a falsely elevated increase in pump flow. In contrast, inflow and outflow thrombosis demonstrates low pulsatility, low flow, and a drop in power as it requires less energy to maintain the set pump speed with less blood flow through the device, as demonstrated by a drop in pulsatility on the HVAD waveform and a decrease in flow.

Given the implications and severity of pump thrombosis, many clinicians have sought to find effective strategies to detect and treat pump thrombosis. Laboratory tests such as LDH and haptoglobin to detect hemolysis along with patient hemodynamics, elevated VAD power consumption, and acoustic analysis have been utilized to detect platelet thrombosis [6]. Imaging modalities such as computed tomography angiography (CTA) and intravascular ultrasound (IVUS) may also assist in the diagnosis of pump thrombosis. A recent study showed that luminal narrowing found on CTA in patients with LVAD outflow grafts was suggestive of extrinsic compression of the graft rather than intraluminal thrombus [7].

Despite advancement in the detection of pump thrombosis, there has not been a consensus on treatment modality. Treatment options such as medical therapy with thrombolytics or surgical device exchange are common; however, the variability of patient and device factors has caused the ideal treatment to be elusive. Studies suggested that the medical treatment of pump thrombosis has a low success rate and a high risk of hemorrhagic stroke and death [8]. It was found that treatment with tPA is more likely to be successful in thrombi that showed gradual development and have not reached a high percent of expected power [9]. LVAD device exchange has shown to have very low early mortality and low complication rates [10].

As the heart failure population grows and LVAD utilization increases, it is of upmost importance that HVAD waveforms are utilized and recognized by heart failure cardiologists, cardiothoracic surgeons, and intensivists to formulate a differential diagnosis to troubleshoot HVAD waveform abnormalities.
